# Regulation of microRNAs in high-fat diet induced hyperlipidemic hamsters

**DOI:** 10.1038/s41598-020-77539-4

**Published:** 2020-11-25

**Authors:** Teodora Barbalata, Lu Zhang, Madalina D. Dulceanu, Camelia S. Stancu, Yvan Devaux, Anca V. Sima, Loredan S. Niculescu, EU-CardioRNA COST Action CA17129

**Affiliations:** 1 Lipidomics Department, Institute of Cellular Biology and Pathology “Nicolae Simionescu” of the Romanian Academy, 8, B. P. Hasdeu Street, 050568 Bucharest, Romania; 2 Cardiovascular Research Unit, Luxembourg Institute of Health, 1445 Strassen, Luxembourg; 3 Present Address: Synevo Romania, 81, Pache Protopopescu Ave, 021408 Bucharest, Romania

**Keywords:** Computational biology and bioinformatics, Molecular biology

## Abstract

Dyslipidemia is a documented risk factor for cardiovascular diseases and other metabolic disorders. Therefore, the analysis of hyperlipidemia (HL)-related miRNAs is a potential approach for achieving new prognostic markers in lipid-metabolism related diseases. We aimed to analyze specific distribution of miRNAs in different tissues from HL animals. Golden Syrian hamsters were fed either regular chow (NL) or high-fat diet (HL) for 12 weeks. Microarray miRNAs profiling was performed in liver, heart and small intestine and data analyzed by R-studio software. Functional enrichment bioinformatics analysis was performed using miRWalk and DAVID tools. We observed a dysregulation of miRNAs in HL tissues evidencing a discrete distribution in the heart-liver axis and three lipid metabolism-related miRNAs were identified: hsa-miR-223-3p, hsa-miR-21-5p, and hsa-miR-146a-5p. Expression levels of these miRNAs were increased in HL livers and hearts. Functional bioinformatics analysis showed involvement of these miRNAs in the regulation of biological processes altered in HL conditions such as lipid metabolic process, fat cell differentiation, regulation of smooth muscle cells and cardiac septum development. We identified a set of miRNAs dysregulated in different tissues of HFD-induced HL hamsters. These findings motivate further studies aiming to investigate novel molecular mechanisms of lipid metabolism and atherogenic HL.

## Introduction

The leading cause of cardiovascular diseases (CVD) is atherosclerosis and its complications, while CVD morbidity and mortality increases annually, despite of published data from many research studies and constantly improving therapeutic approaches. Atherosclerosis is known as an inflammatory disease that is generated and/or aggravated by lipid metabolism disorders^1,2^. Early CVD diagnosis and detection of new molecular targets may help introduce early therapeutic interventions to improve the CVD outcomes.

The ENCODE pilot project indicated that most of the genome transcripts that have no protein-coding potential are actually RNA molecules with functional activity and can regulate pathophysiological tissue homeostasis^3^. These non-coding transcripts appeared as major regulators of CVD^4^. Epigenetic regulation of lipid metabolism at transcriptional and post-transcriptional level earned growing interest^5^. Among other epigenetic factors, microRNAs (miRNAs) are strong post-transcriptional gene regulators, also known to control the expression of lipid metabolism-related genes^6^. Moreover, miRNAs are found in various tissues and in the bloodstream, their circulating levels being proposed as potential biomarkers to diagnose cardiovascular events, like acute myocardial infarction (AMI)^7^. Hyperlipidemia is a known risk factor for CVD progression^8,9^, whose regulation is also known to be resolved by miRNA-mediated mechanisms. Therefore, discovering specific hyperlipidemia-associated miRNAs could be a feasible approach for designing either miRNA-based therapies or achieving new prognostic markers in lipid metabolism-related disorders.

The Golden Syrian hamster (*Mesocricetus auratus*) presents many similarities to the human lipid metabolism, being outfitted with cholesterol esters transfer protein, peroxisome proliferator-activated receptors, and all of the enzymatic mechanisms participating to lipoproteins and bile metabolism that are also present and active in humans^10–15^. We have previously reported that high fat diet (HFD)-fed hamsters presented mixed hyperlipidemia and developed coronary atherosclerosis^11,16^. We here hypothesize that the changes in miRNAs expression in the HFD-induced hyperlipidemic (HL) hamster could contribute to alterations of the lipid metabolism. Identifying miRNAs regulated during HL development would allow gaining insight into the molecular mechanisms implicating miRNAs and responsible for atherogenesis under hyperlipidemic conditions. Hence, the aim of study was to analyze the distribution of miRNAs in different tissues of HFD-induced HL hamsters compared to normolipidemic hamsters using microarrays and functional bioinformatics tools.

## Results

### Discovery study—the different expression profiles of miRNAs regulated in HL hamsters

**Figure 1:**
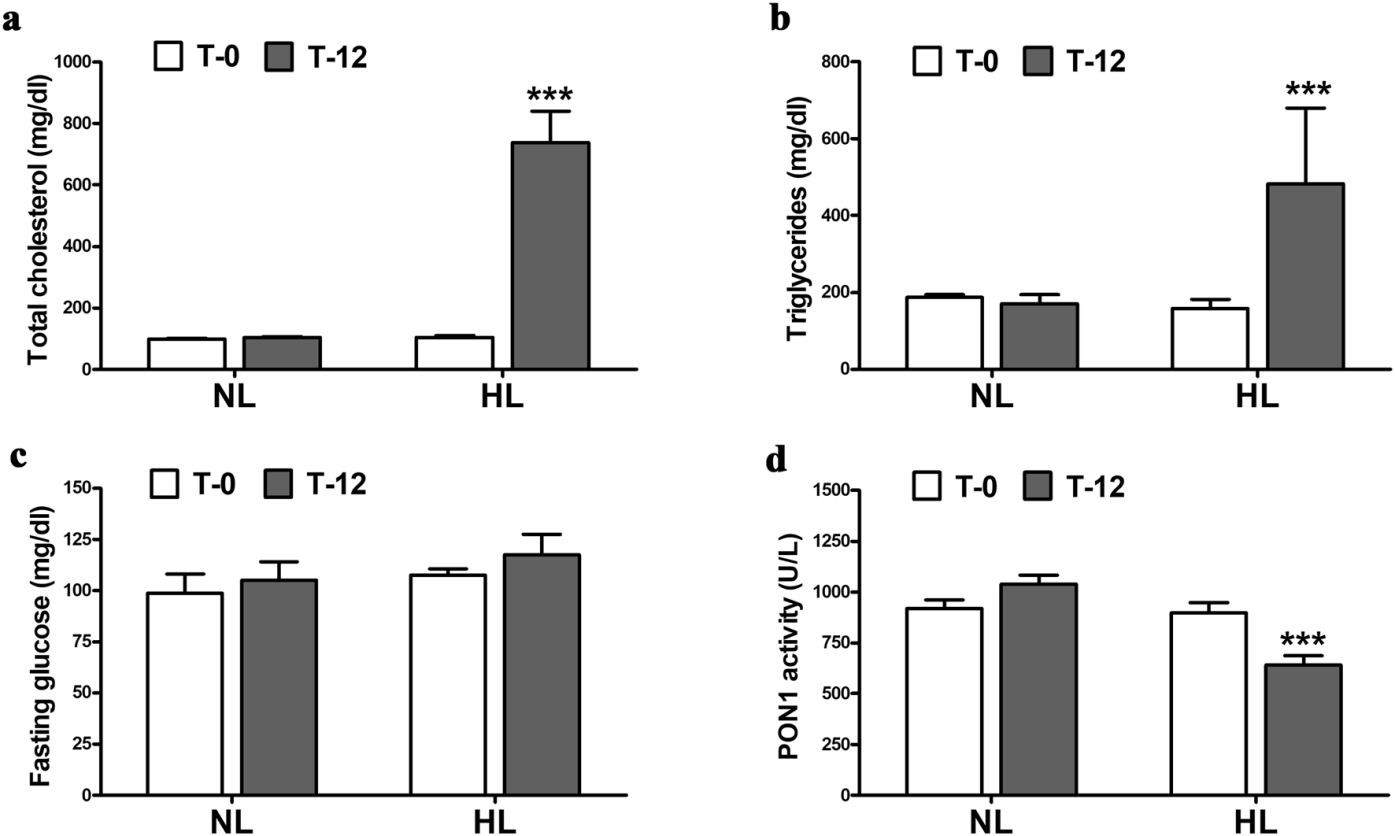
Plasma levels of total cholesterol (**a**), triglycerides (**b**), fasting glucose (**c**) and paraoxonase (PON1) activity (**d**) in normolipidemic (NL) hamsters and high-fat diet (HFD)-induced hyperlipidemic (HL) hamsters from the *discovery group*, at beginning (T-0) and after 12 weeks (T-12) of diet. ***p < 0.001 vs T-0 (n = 5 animals per group, Mann–Whitney U test).

As we have previously reported^17^, the hamsters presented combined hypercholesterolemia and hypertriglyceridemia after 12 weeks of HFD (T-12), showing about sevenfold higher total cholesterol, 2.8-fold higher triglycerides plasma levels compared with T-0 and to normolipidemic (NL) hamsters (p < 0.001) (Fig. 1a,b). Glucose levels in plasma of HL hamsters did not vary after 12 weeks of HFD compared with T-0 and to NL hamsters (Fig. 1c). An increased oxidative stress was detected in plasma of HL hamsters, expressed as about 40% reduction in paraoxonase 1 (PON1) activity (Fig. 1d).

**Figure 2:**
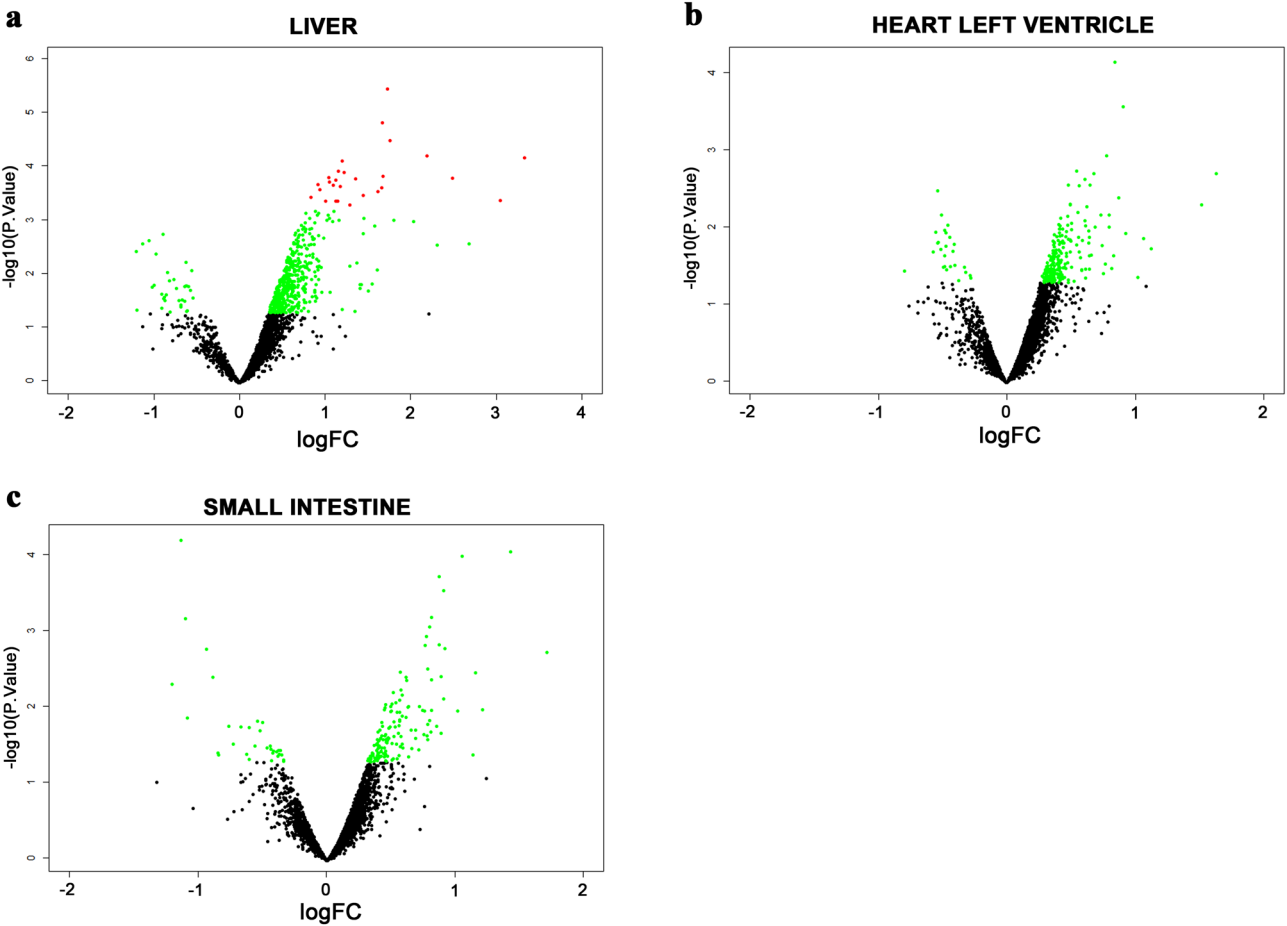
Volcano plots of miRNAs expression profiles in right lobe of the liver (**a**), left ventricle of the heart (**b**) and small intestine (**c**) from hyperlipidemic hamsters normalized to normolipidemic hamsters. The green spots (•) represent miRNAs with p < 0.05 and the red spots (•) depict miRNAs with p < 0.05 and FDR < 0.05.

**Figure 3:**
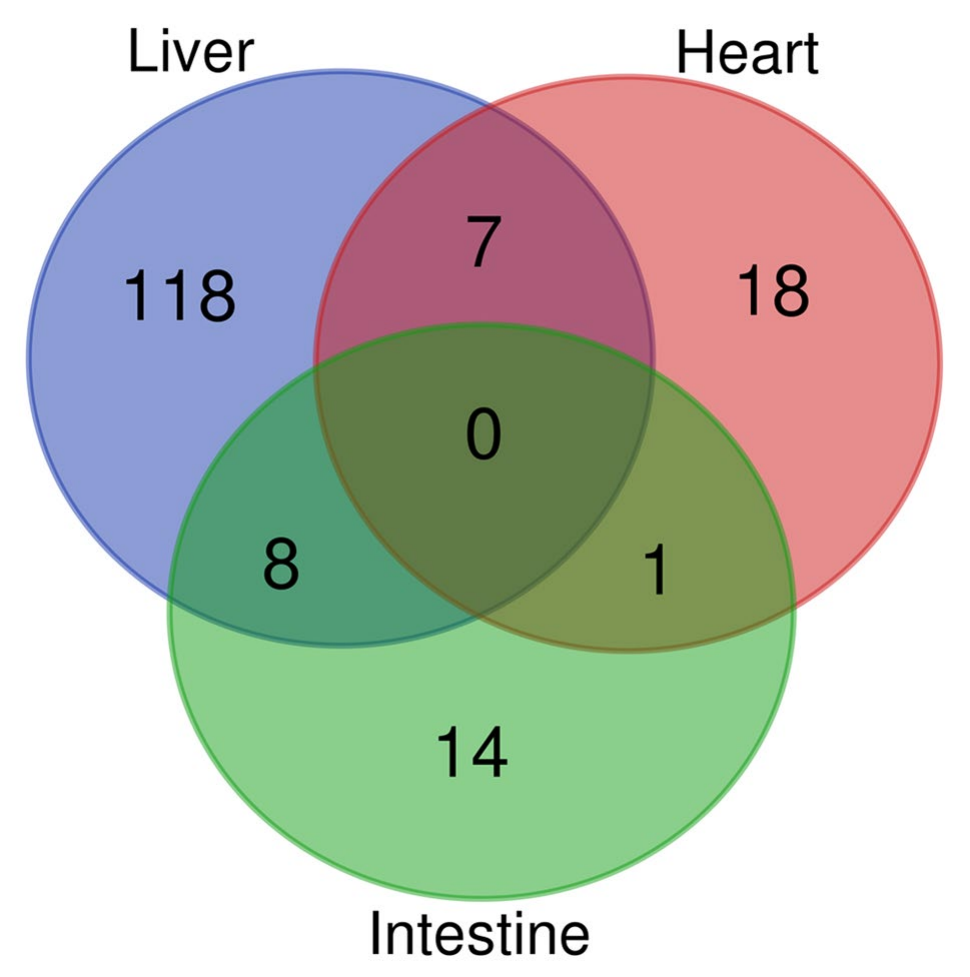
The comparison of differentially expressed (p < 0.05 and FC ≥ 1.5) miRNAs with human homologs in right lobe of the liver, left ventricle of the heart and small intestine. Detailed list of shared miRNAs is shown in Supplementary Table S4.

We then performed the microarray using the RNAs isolated from the liver, the heart and the intestine of NL and HL hamsters. Figure 2 presents miRNA expression profiles from different tissues of HL hamsters compared to NL hamsters. The expression level of 286, 38 and 60 miRNA probes, including 134, 26 and 23 human miRNAs, on the microarray were changed in the liver, the heart and the intestine respectively of HL hamsters with p < 0.05 and fold-change (FC) ≥ 1.5 (Supplementary Tables S1–S3). We did not find any miRNAs with human homologues that were commonly differentially expressed in the three tissues. Seven miRNAs were commonly differentially expressed in the liver and the heart, 8 were common in the liver and the intestine, only one common miRNA in the heart and the intestine (Fig. 3 and Supplementary Table S4).

We observed that 35, 10 and 3 miRNAs with human homologs from the liver, the heart and the intestine respectively were conserved in mouse and/or rat. Among them, hsa-miR-146a-5p was increased in both the liver (FC = 2.92 and p = 0.014) and the heart (FC = 1.79 and p = 6.84 × 10^–5^) of HL hamsters. Hsa-miR-146a-5p and hsa-miR-223-3p (FC = 1.56, p = 0.0018) were the 2 most altered miRNA in HL hearts, after ranking by p values. In the liver of HL hamsters, hsa-miR-21-5p was the most differentially expressed conserved miRNA (ranked by p values), having FC = 10.03 and p = 6.46 × 10^–5^. These three miRNAs were retained for further validation, the selection being done according to their expression changes in HL hamsters’ tissues and based on our previous data from human subjects. Accordingly, the circulating levels of hsa-miR-146a-5p and hsa-miR-223-3p have been previously reported associated with hyperlipidemia and disease vulnerability in coronary artery disease (CAD) patients^18,19^, while hsa-miR-21-5p levels has been found specifically increased in sera of hyperlipidemic subjects^20^.

### Validation study—qPCR confirmation of selected miRNAs in liver and heart

Three selected miRNAs were further validated by measuring their expression levels in tissues (liver, heart and small intestine) from HL hamsters fed HFD for 21 weeks (*validation study* group). As we previously reported^16^, HL hamsters presented mixed hyperlipidemia after 21 weeks of HFD, having 19-fold higher total cholesterol levels and 15-fold higher triglycerides levels in plasma than normal ranges measured in NL hamsters. We chose to perform microarray analysis for the identification of lipid metabolism-related miRNAs in hamsters after 12 weeks HFD and then to validate them at 21 weeks HFD in order to highlight those miRNAs that have modified expression in the advanced stages of atherosclerosis, but whose levels start to show changes earlier, with respect to the onset of the disease.

**Figure 4:**
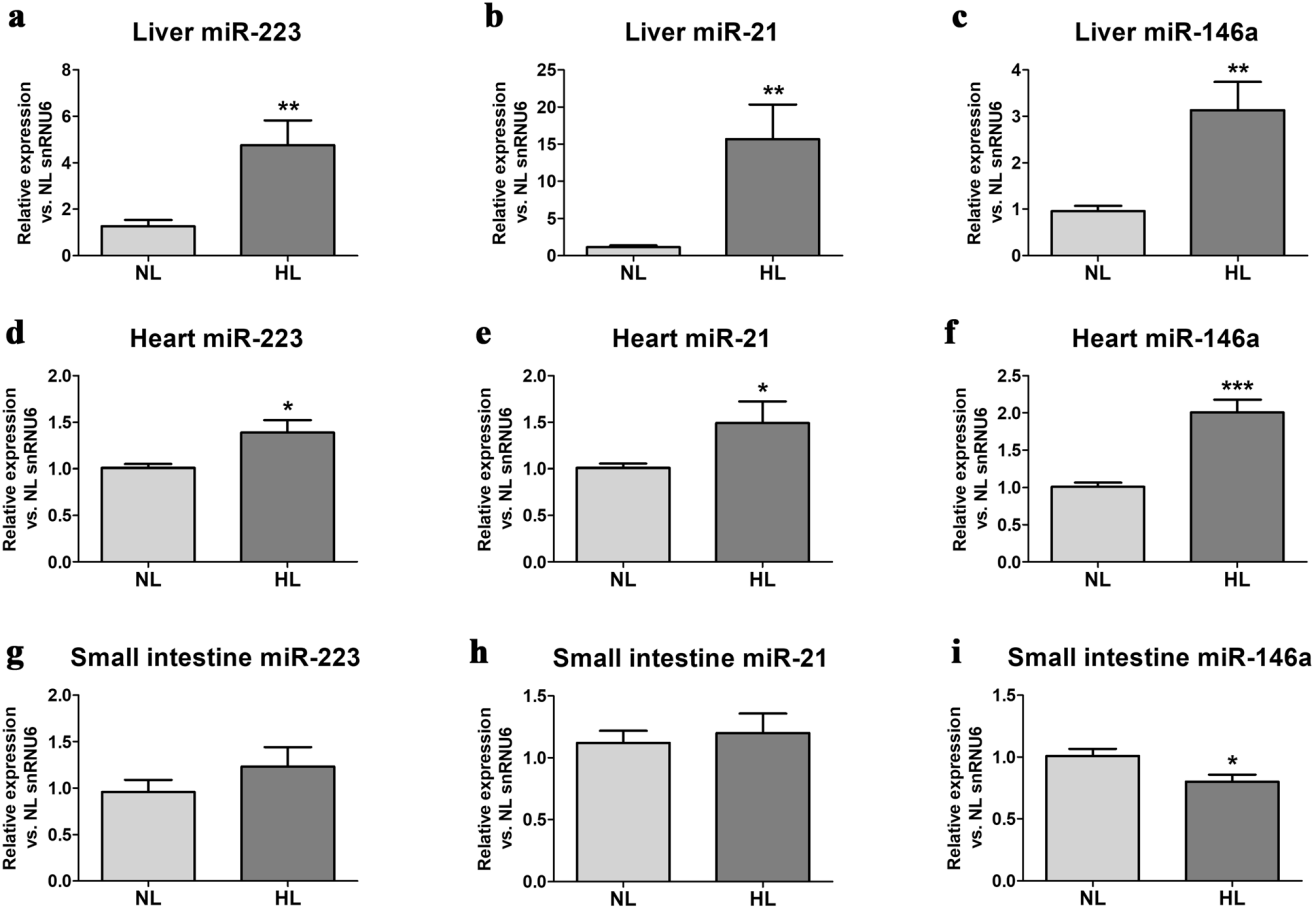
Expression levels of selected miRNAs (miR-223, miR-21 and miR-146a) in liver (**a**–**c**), left ventricle of the heart (**d**–**f**), and small intestine (**g**–**i**) from normolipidemic (NL) hamsters and high-fat diet (HFD)-induced hyperlipidemic (HL) hamsters from the *validation* group after 21 weeks of diet. *p < 0.05, **p < 0.01, ***p < 0.001 vs NL group (n = 10 animals per group, Mann–Whitney U test).

Expression levels of the three miRNAs significantly increased in the liver of HL hamsters compared to NL group: miR-21-5p (15.7-fold, p < 0.001), miR-223-3p (4.7-fold, p < 0.001) and miR-146a-5p (threefold, p < 0.001), confirming the microarray data of the discovery study (Fig. 4a–c). Similarly, the levels of the three miRNAs were enhanced in the LV of hearts from HL hamster compared to NL group: miR-146a-5p (twofold, p < 0.001), miR-21-5p (1.5-fold, p < 0.05) and miR-223-3p (1.4-fold, p < 0.05), consistently with the microarray data (Fig. 4d–f). In contrast, in the small intestine of HL hamsters, miR-146a-5p decreased significantly (by 20%, p < 0.05), while the other two miRNAs showed no significant variation compared to NL hamsters (Fig. 4g–i).

### Functional analysis—biological processes targeted by the three miRNAs set

**Figure 5:**
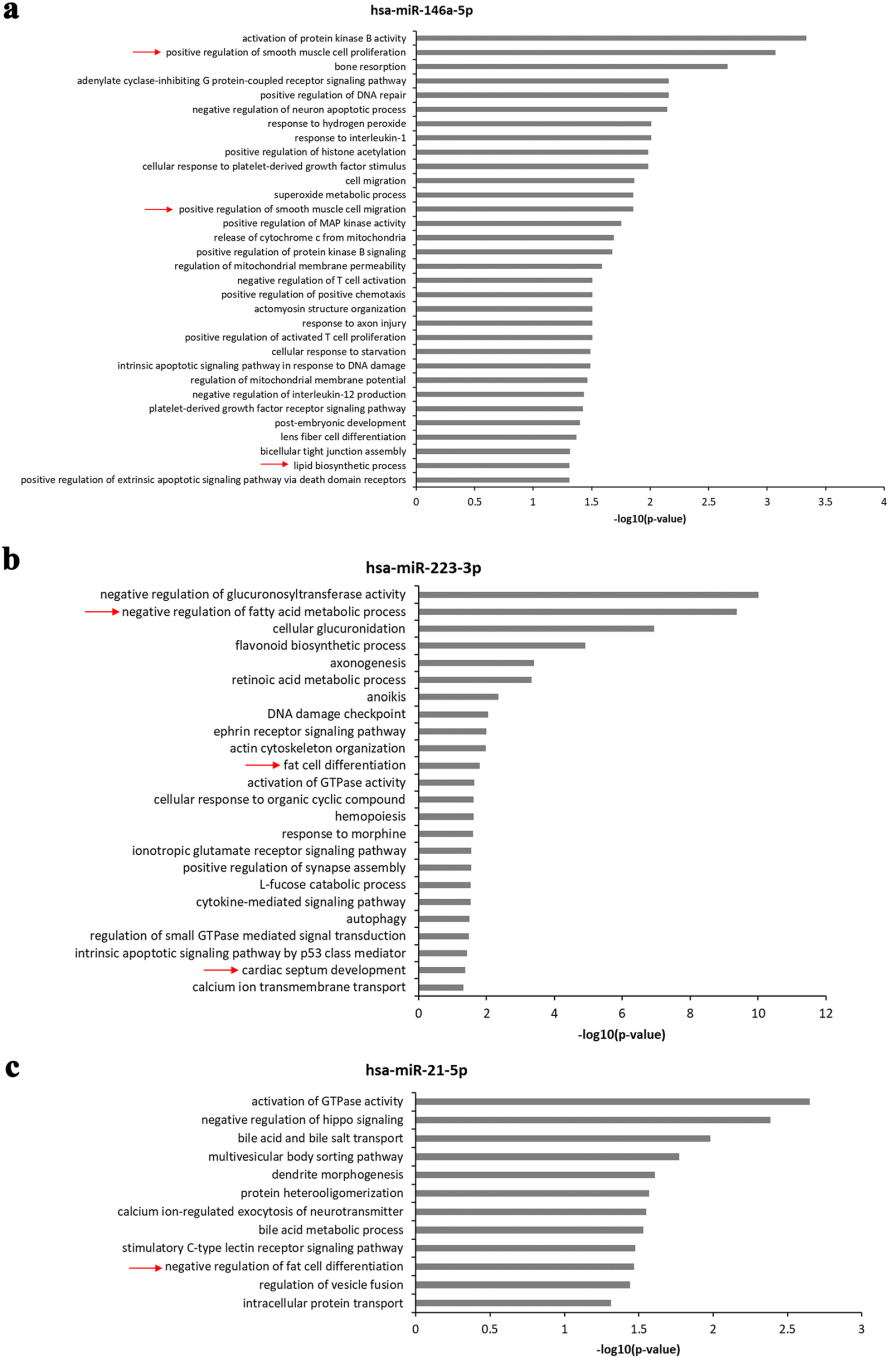
DAVID functional analysis for genes identified by miRWalk algorithm to be potentially targeted by the selected miRNAs i.e. miR-146a-5p (**a**), miR-223-3p (**b**) and miR-21-5p (**c**). The red arrows (**→**) show the metabolic processes known to be altered in dyslipidemia and atherosclerosis.

The miRWalk predicted 506, 332 and 554 human genes potentially targeted by hsa-miR-146a-5p, hsa-miR-21-5p and hsa-miR-223-3p respectively. Only two genes, NTRK3 and KLHL18, were common throughout the targets of the 3 miRNAs. We performed biological process enrichment analysis for the target genes of each miRNA using DAVID. 32 biological processes were found implicated by the targets of hsa-miR-146a-5p, which was up-regulated in the heart and the liver of HL hamsters (Fig. 5a), and two of them were associated with the positive regulation of smooth muscle cells proliferation and migration (Fig. 5a). These targets were also enriched in lipid biosynthetic process (Fig. 5a). The targets of hsa-miR-223-3p, which was up-regulated in the heart of HL hamsters, were found enriched in 24 biological processes including negative regulation of fatty acid metabolic process, fat cell differentiation and cardiac septum development (Fig. 5b). The targets of hsa-miR-21-5p, which was up-regulated in the liver of HL hamsters, were associated with 12 biological processes including negative regulation of fat cell differentiation (Fig. 5c).

## Discussion

In this study, we characterized tissue-specific miRNA signatures in different tissues of HL hamsters. We identified and validated a set of lipid metabolism-related miRNAs (hsa-miR-223-3p, hsa-miR-146a-5p, and hsa-miR-21-5p) with dysregulated expression in HL hamster tissues. Bioinformatic analysis predicted the biological processes that are potentially regulated by this set of miRNAs, and among them we highlighted regulation of lipid metabolic process, fat cell differentiation, regulation of smooth muscle cells function and cardiac septum development. These data are in good agreement with our previous data on CAD patients and HFD-induced HL hamsters. We have previously demonstrated that miR-223 and miR-146a are specifically increased in plasma and HDL fractions from vulnerable CAD patients^18,19^. Also, we showed that miR-223 levels are augmented in plasma and livers from HFD-fed hamsters^17,21^. In other studies, we showed that hsa-miR-146a-5p and hsa-miR-21-5p are specifically associated with hyperlipidemia, being correlated with inflammatory parameters in HL patients^20^. All these data raise the possibility of an alternative use of the in vivo inhibition of miRNAs to assist the statin-based HL management in humans by correcting the dysregulated lipid metabolism and modulating the associated atherosclerotic process. However, the potential translation of the HL hamster data to humans lacks the knowledge on how the lipid metabolism is regulated at the transcriptional and post-transcriptional level, due to the absence of a complete hamster genome sequencing.

To date, there are only few published studies profiling the transcriptome in Golden Syrian hamsters^22,23^, but the reported RNA sequencing analysis was performed in a high fructose-fed model, designed for the study of very low density lipoproteins (VLDL) assembly association with insulin resistance. The fructose-fed hamster displays typical whole body insulin resistance with marked hepatic VLDL and triglycerides overproduction, which was set up as an ideal model for investigating VLDL metabolism in insulin resistance^24^. The group of Li et al. performed deep sequencing and constructed an mRNA-miRNA-lncRNA interaction network of the fructose-fed hamster liver to find potential RNA molecular regulation of the VLDL production^22^. They reported 146 differentially expressed coding genes, 27 differentially expressed lncRNA genes, as well as 16 differentially expressed miRNAs^22^. In another study, the group of Sud et al. performed computational prediction and functional analysis and thus identified ten miRNAs assembled as a regulatory network to potentially target key genes in lipid and lipoprotein metabolism and insulin signaling pathway at multiple levels^23^. These studies revealed that the miRNA profile induced by high-fructose diet differed from that induced by HFD, indicating that miRNAs mediate distinct pathogenic mechanisms in dietary-induced metabolic disorders.

Among the here identified and validated miRNAs, miR-223 is remarkable because of its proven role in the regulation of cholesterol metabolism^25^. According to Vickers’ results, the target genes of miR-223 are the hydroxyl-methyl-glutaryl-CoA synthase 1 and the scavenger receptor class B type I, two key genes involved in the cholesterol synthesis, and respectively, in the cholesterol efflux process in mammalian cells^25^. We demonstrate here that miR-223 expression is specifically increased in liver and heart from HFD-induced HL hamsters. In good agreement with these data, we have previously shown that probiotics treatment could reduce the HL-associated increased serum and hepatic lipids, miR-223 levels, and the expression of the miRNAs’ processing proteins (Dicer, DGCR8) in the livers of HL hamsters^21^. Wang’s group reported that miR-223 reduces lipid accumulation in cultured macrophages by activating phosphatidylinositol 3-kinase/protein kinase B pathway, suggesting a direct connection between miR-223 and the cholesterol metabolism^26^. These results obtained in HL hamsters confirm our previous data demonstrating that plasma and high density lipoproteins (HDL)-associated miR-223 levels are increased in hyperlipidemic and/or hyperglycemic CAD patients^19^.

It is known that the expression of miR-146a is increased in human atherosclerotic plaques^27^, and this could happen probably as a protective feedback mechanism, being known that miR-146a overexpression downregulates the nuclear factor κ-B that in turn reduces atherosclerosis^28^. We have previously investigated the expression of miR-146a in the sera of CAD patients based on the reports showing that these miRNAs are related to cardiac injury or myocyte cell death^29^. Consistently with our previous results in CAD patients^18^ and with the here presented data in livers and hearts from HL hamsters, circulating levels of miR-146a were reported to be markedly elevated in ACS patients compared to non-ACS patients^30^. An unexpected decrease in intestinal miR-146a was also observed in 21-week HFD-fed hamsters compared to NL littermates, and this could be explained by the difference between the *discovery* (12-week HFD) and *validation* (21-week HFD) groups, the latter being developed as a model for the study of advanced stages of atherosclerosis.

Recently, miR-21 gained special interest due to its role in the regulation of many biological and pathological processes, mainly in CVD. Altered expression of miR-21 was reported in diseases like CVD, inflammation-induced diseases, or oncological diseases, and it is also involved in immunological and developmental processes^31^. The measured increase of miR-21 in HL hamsters’ tissues is consistent with Zhang et al. data showing significantly up-regulated miR-21-5p in plasma and aorta from HFD-fed rats^32^. In mice, published data show that miR-21 modulates vascular remodeling by regulating TGF-β signaling, but its target genes involved in this process are not known^33^. MiR-21 has higher expression levels in hypertrophied mice hearts^34^. Recent data showed that alterations of miR-21 tissue expression due to AMI may depend on AMI moment and specific location within the heart. Accordingly, it was reported that miR-21 expression is down-regulated in infarcted areas of the heart, but up-regulated in borderline areas^35^. Additional data show that miR-21 might have an important regulatory role in the pathophysiology of AMI and could have cardioprotective effects most likely by inhibiting apoptosis^36^. However, published data on the role of circulating miR-21 levels during post-AMI are disputed and, therefore, miR-21 could not be proposed yet as a potential biomarker of AMI.

A potential limitation of our study is that animals from the *discovery* group received HFD for 12 weeks, while a HFD period of 21 weeks was given to those from the *validation* group. After 12 weeks of HFD administration, hamsters acquire steady hyperlipidemia without developing life threatening atheroma. These advanced lesions became evident in animals after 20 weeks of HFD intake^16^. We chose to identify lipid metabolism-related miRNAs by microarray analysis at 12 weeks HFD and then to validate them in hamsters at 21 weeks HFD in order to emphasize those miRNAs that are altered in the advanced stages of atherosclerosis, but which start to show changes in their levels since the onset of the disease. In this way, the highlighted miRNAs might be able to serve as early candidate for the role of predictive biomarkers in CVD.

In conclusion, we report a HL-specific profile of miRNAs in different hamster tissues. Expression levels of a set of three miRNAs (hsa-miR-223-3p, hsa-miR-146a-5p and hsa-miR-21-5p) were increased in HFD-induced HL hamster tissues. In silico functional analyses suggested the specific involvement of these miRNAs in the regulation of biological processes altered in HL conditions. These findings encourage further studies investigating novel molecular mechanisms of lipid metabolism and atherogenesis.

## Methods

### Experimental animals

For the *discovery study*, 10 male Golden Syrian hamsters (*Mesocricetus auratus*, 12–14 weeks old, averaged body weight 123 ± 7.4 g) were kept under standard housing conditions (two–three per cage, 12 h light/dark cycles) with ad libitum access to water and standard rodent chow for 12 weeks—NL group (n = 5 hamsters) or high-fat diet (HFD) involving standard rodent chow supplemented with 3% cholesterol and 15% butter—HL group (n = 5 hamsters). For the *validation study*, 20 male hamsters were divided in NL (n = 10) and normoglycemic HL (n = 10) group fed either regular chow (NL group) or HFD (HL group) for 21 weeks.

At the inception of HFD and after 12 or 21 weeks, all hamsters were anesthetized (with 4–5% isoflurane in oxygen flow) and a minimum volume of blood (~ 300 μL) was collected on EDTA from the venous retro-orbital plexus after overnight fasting. Plasma was separated and analyzed immediately or stored at − 80 °C until analysis. NL and HL hamsters after overnight fasting were subjected to laparotomy under lethal ketamine-xylazine (50–5 mg Kg^−1^ body) anesthesia and the vasculature was cleared by perfusion with warm phosphate buffered saline (PBS) for 5 min (inlet, left ventricle; outlet, right atrium). The liver, myocardium and small intestine (jejunum) were collected, PBS-cleaned, frozen in liquid nitrogen and stored at − 80 °C until RNA analysis. Before the procedure, blood was collected on EDTA from the venous retro-orbital plexus (under 4–5% isoflurane anesthesia) for biochemical analysis.

All the procedures performed on experimental animals were carried out in accordance with the EU Directive 2010/63/EU on the protection of animals used for scientific purposes, and have the approval from the Romanian Sanitary Veterinary and Food Safety Authority (permission no. 256/23.05.2016).

### Biochemical analyses in plasma

The fasting glucose and lipids (total cholesterol and triglycerides) levels in hamsters’ plasma were assessed by using commercial kits (Dialab, Neudorf, Austria), while paraoxonase (PON1) activity was quantified with an adapted protocol^37^.

### Microarray analysis for specific miRNAs distribution in hamster’ tissues

Individual tissue samples (~ 20 mg frozen tissue) from the right lobe of the liver, left ventricle of the heart and small intestine—jejunum from three randomly selected NL and HL hamsters from the discovery group were processed for total RNA isolation using a Silent Crusher M homogenizer (Heidolph Instruments GmbH, Schwabach, Germany) and Trizol reagent (Thermo Scientific, Waltham, MA, USA). RNA quality was controlled by use of Agilent 2100 bioanalyzer (Agilent Technologies Inc., Santa Clara, CA, USA) and Nanodrop Lite Spectrophotometer (Thermo Scientific). A fixed quantity of 750 ng from each sample was blended with the Spike-in miRNA kit (Exiqon, Denmark) and labeled using Exiqon miRCURY LNA microRNA Hi-Power Labeling Kit. We used the single color (Hy3) labeling protocol for the miRCURY LNA microRNA Array, 7th generation kit for human, mouse and rat miRNAs, miRBase v19 (Exiqon, Denmark). According to the manufacturer data, over 72% of hamster (*Crisetulus griseus*) miRNAs sequences from miRBase v19 are compatible with microarray 7th generation design. All the procedures were done accordingly to manufacturer's protocol. Calf intestinal alkaline phosphatase (CIP) treatment and labeling were done on a Veriti PCR machine (Applied Biosystems, USA) and hybridization was performed using hybridization cassettes, gaskets and hybridization oven from Agilent Technologies, USA. Immediately after washing and drying, the array slides were visualized using the Innoscan 1100AL scanner with Mapix software (Innopsys, France) at 10 μm resolution, green laser power (532 nm, 65% gain, set at high). The initial raw data were obtained after image processing with Imagene software (Innopsys, France) using the indicated gal files from Exiqon. Limma R package was used for expression analysis. Briefly, raw data was pre-processed with the local background correction followed by the quantile normalization between the microarrays. A linear model was fitted to normalized data. We used empirical Bayes statistics for differential expression and Benjamini & Hochberg method (FDR) to adjust p-value. A Venn diagram was constructed using an online tool (http://bioinformatics.psb.ugent.be/webtools/Venn) to compare the miRNAs regulated in different hamster’s tissues. Both microarray raw data and log2 transformed normalized signal intensity can be accessed in GEO database with accession number GSE128226.

### Measurement of miRNAs levels in hamsters’ tissues

Total RNA was extracted from hamsters’ tissues with TRIzol reagent (Thermo Sci., USA), according to the manufacturer’s protocol, resuspended in 100 μL of RNase-free water and stored at − 80 °C. All the analyzed miRNAs are conserved between human and hamster (*Crisetulus griseus*). The tissue expression of hsa-miR-223-3p (ID 002295), hsa-miR-21-5p (ID 000397), and hsa-miR-146a-5p (ID 000468) were measured by using TaqMan technology (Thermo Sci., USA) as we previously reported, normalizing against snRNU6 (ID 001973)^17,21^.

### Functional analysis of miRNAs

The miRNA targets were predicted using the online tool miRWalk v.3 (mirwalk.umm.uni-heidelberg.de)^38^. DAVID (The Database for Annotation, Visualization and Integrated Discovery) platform was used for biological process enrichment analysis^39^. The biological process terms with p value < 0.05 and fold enrichment > 2 were accounted for significance. Biological process level was defined as the depth of node in AmiGO2 inferred tree view (the lower level, the more general)^40^. The depth of biological process was set to 0 and those terms deeper than 3 were considered relevant. Child annotation terms in the same branch were discarded to avoid redundancy.

### Statistical analysis

GraphPad Prism 6.0 (GraphPad Software Inc., San Diego, CA, USA) software was used for the statistical analysis of lipids and tissue miRNAs data in hamsters. The continuous distributed quantitative variables (biochemical and miRNAs data) were expressed as means ± standard error of the mean (SEM) and analyzed by Mann–Whitney U test. The threshold for statistical significance was set for p-values lower than 0.05.

## Supplementary information

Supplementary Information

## Data Availability

Microarray raw data and log2 transformed normalized signal intensity can be accessed in GEO database with accession number GSE128226. We state that the materials, data and associated protocols used in the present work will be made promptly available to readers without undue qualifications in material transfer agreements. We disclose no restriction on the availability of materials or information in this manuscript.
