# Supplementary material for: Regulation of microRNAs in high-fat diet induced hyperlipidemic hamsters
Source: Sci Rep. 2020 Nov 25;10:20549. doi: 10.1038/s41598-020-77539-4 (PMC7688633; doi:10.1038/s41598-020-77539-4)
Supplement: Supplementary file 1 — Supplementary Information [file 41598_2020_77539_MOESM1_ESM.pdf]

Supplementary information

Article:

Regulation of microRNAs in high-fat diet induced hyperlipidemic hamsters

Authors:

Teodora Barbalata [1,#], Lu Zhang [2,#], Madalina D. Dulceanu [1,†], Camelia S. Stancu [1], Yvan Devaux [2], Anca V. Sima [1], and Loredan S. Niculescu [1,*], on behalf of EU-CardioRNA COST Action CA17129

[1] Lipidomics Department, Institute of Cellular Biology and Pathology "Nicolae Simionescu" of the Romanian Academy, 8, B. P. Hasdeu Street, Bucharest 050568, Romania

[2] Cardiovascular Research Unit, Luxembourg Institute of Health, L-1445 Strassen, Luxembourg

[#] These authors contributed equally to this work.

[†] Current address: Synevo Romania, 81, Pache Protopopescu Ave., Bucharest 021408, Romania

*** Corresponding author:**

Loredan S. Niculescu, Ph.D.

Lipidomics Department, Institute of Cellular Biology and Pathology "Nicolae Simionescu" of the Romanian Academy

8, B. P. Hasdeu Street, 050568 Bucharest, Romania

Phone: +4021 319 4518 / Fax: +4021 319 4519 / E-mail: loredan.niculescu@icbp.ro

Supplementary Table S1.
MiRNAs regulated in the liver (right lobe) of HL hamsters with $p < 0.05$ and fold-change ≥ 1.5, ranked with fold-change.

Gene.ID	Name	Fold-change	Expression level	p-value	FDR
42832	hsa-miR-638	3.3066	10.9283	3.38E-06	0.00453
168637	hsa-miR-3940-5p	3.1868	13.7282	1.42E-05	0.01273
168944	hsa-miR-4707-5p	3.3798	10.4361	3.09E-05	0.02077
147506	hsa-miR-21-5p/mmu-miR-21a-5p/rno-miR-21-5p	10.0325	10.3221	6.46E-05	0.02808
10947	hsa-miR-142-3p/mmu-miR-142a-3p/rno-miR-142-3p	2.3002	12.6336	7.32E-05	0.02808
168814	hsa-miR-4463	2.3380	9.6904	1.19E-04	0.03185
169171	hsa-miR-4436b-5p	3.2039	12.0591	1.43E-04	0.03185
169388	hsa-miR-663a	2.5603	9.8481	1.59E-04	0.03185
168642	hsa-miR-642b-3p	2.0758	9.3435	1.80E-04	0.03229
168675	hsa-miR-4433a-3p	1.8844	8.9910	2.04E-04	0.03260
148217	hsa-miR-23c	2.1397	10.3915	2.07E-04	0.03260
169096	hsa-miR-4804-3p	3.0590	10.3601	2.75E-04	0.03517
168668	hsa-miR-4732-3p	2.7294	12.3595	3.24E-04	0.03956
42446	hsa-miR-576-5p	2.1486	10.9823	6.33E-04	0.05737
168904	hsa-miR-4473	1.8937	11.0769	6.98E-04	0.05880
169130	hsa-miR-4764-3p	2.0699	13.6454	8.54E-04	0.06301
42530	hsa-let-7a-2-3p/mmu-let-7a-2-3p/rno-let-7a-2-3p	2.7350	10.4616	8.68E-04	0.06301
168919	hsa-miR-4456	2.2351	12.5150	9.23E-04	0.06335
169273	hsa-miR-548ar-3p	3.4818	7.3831	9.44E-04	0.06335
168650	hsa-miR-3121-5p	1.8437	9.4941	1.04E-03	0.06358
145768	hsa-miR-665	1.6513	11.2756	1.15E-03	0.06883
46258	hsa-miR-1184	1.7964	9.3472	1.35E-03	0.07275
168702	hsa-miR-4540	1.7661	10.7072	1.36E-03	0.07275
148621	hsa-miR-892a	1.7011	10.6861	1.51E-03	0.07647
19582	hsa-miR-106b-5p/mmu-miR-106b-5p/rno-miR-106b-5p	2.7261	9.5071	1.64E-03	0.07832
42929	hsa-miR-25-5p/mmu-miR-25-5p	1.7681	12.1138	1.66E-03	0.07832
42551	hsa-miR-122-3p/mmu-miR-122-3p	0.5372	13.2574	1.72E-03	0.07832
169322	hsa-miR-4534	1.6703	9.1972	1.74E-03	0.07832
148536	hsa-miR-1-5p/mmu-miR-1a-1-5p	1.5653	9.1329	1.76E-03	0.07832
148247	hsa-miR-2355-3p	1.6134	10.5813	1.81E-03	0.07832
147611	hsa-miR-4305	1.6090	9.4891	1.94E-03	0.08118
168994	hsa-miR-3591-5p	1.9771	12.5450	1.99E-03	0.08229
168870	hsa-miR-1246	1.6756	11.3508	2.20E-03	0.08443
169232	hsa-miR-3156-3p	0.4813	14.7809	2.24E-03	0.08487
42899	hsa-miR-377-5p/mmu-miR-377-5p/rno-miR-377-5p	1.5507	9.8181	2.53E-03	0.08804
147743	hsa-miR-4275	0.4573	13.6027	2.54E-03	0.08804
17498	hsa-miR-601	1.5778	9.8771	2.58E-03	0.08804
147767	hsa-miR-4279	1.6030	12.1873	2.58E-03	0.08804

145905	hsa-miR-518a-5p/hsa-miR-527	1.7838	9.1374	2.63E-03	0.08804
11104	hsa-miR-422a	1.8326	9.9715	2.65E-03	0.08804
147186	hsa-miR-200b-3p/mmu-miR-200b-3p/rno-miR-200b-3p	1.6924	9.9468	2.71E-03	0.08804
46737	hsa-miR-1265	1.6482	9.4821	2.93E-03	0.09037
169381	hsa-miR-4421	1.7217	12.3393	3.09E-03	0.09435
169266	hsa-miR-378d	1.7251	10.2264	3.23E-03	0.09645
147614	hsa-miR-4299	1.5346	11.8033	3.53E-03	0.10266
169399	hsa-miR-4750-5p	1.5528	9.8875	3.87E-03	0.10802
169045	hsa-miR-4651	1.5253	8.7629	4.02E-03	0.10802
147706	hsa-miR-4255	1.5412	9.8356	4.18E-03	0.11015
148668	hsa-miR-378a-3p/mmu-miR-378a-3p/rno-miR-378a-3p	1.7204	11.3694	4.35E-03	0.11229
42475	hsa-miR-221-5p/mmu-miR-221-5p/rno-miR-221-5p	1.8142	8.4300	4.61E-03	0.11669
169313	hsa-miR-4800-3p	1.5741	8.8913	5.32E-03	0.12077
46731	hsa-miR-4657	1.7025	11.9816	5.53E-03	0.12077
42744	hsa-miR-23a-3p/mmu-miR-23a-3p/rno-miR-23a-3p	2.5915	9.5688	5.87E-03	0.12077
168928	hsa-miR-4431	1.5037	9.2931	5.94E-03	0.12080
168863	hsa-miR-4441	1.5258	10.6437	6.20E-03	0.12201
148068	hsa-miR-758-5p/mmu-miR-758-5p/rno-miR-758-5p	1.6016	10.4898	6.31E-03	0.12201
169312	hsa-miR-548an	1.6036	10.2571	6.59E-03	0.12201
147904	hsa-miR-3148	1.6742	10.2603	6.63E-03	0.12201
145987	hsa-miR-2054	1.7205	8.4565	6.65E-03	0.12201
147820	hsa-miR-3133	1.6436	11.6880	6.81E-03	0.12201
27565	hsa-miR-423-5p/mmu-miR-423-5p/rno-miR-423-5p	1.5186	10.8125	6.95E-03	0.12201
29575	hsa-miR-32-3p/mmu-miR-32-3p/rno-miR-32-3p	1.8869	12.5069	6.98E-03	0.12201
10977	hsa-miR-183-5p/mmu-miR-183-5p/rno-miR-183-5p	1.7559	8.6436	7.90E-03	0.12548
46380	hsa-miR-1255a	1.8841	12.8856	7.97E-03	0.12548
42476	hsa-miR-374b-3p	1.5610	9.1778	8.01E-03	0.12548
168819	hsa-miR-200a-3p/mmu-miR-200a-3p/rno-miR-200a-3p	1.5843	9.7136	8.09E-03	0.12548
168653	hsa-miR-3158-5p	1.6623	10.4006	8.61E-03	0.13036
148652	hsa-miR-620	1.5149	10.7940	9.46E-03	0.13715
148495	hsa-miR-3915	1.6036	11.8076	9.66E-03	0.13715
168727	hsa-miR-4426	1.5362	10.3900	9.79E-03	0.13715
169264	hsa-miR-4762-5p	1.5273	10.4619	9.83E-03	0.13715
169305	hsa-miR-4455	1.8529	12.7260	0.01020	0.13715
147806	hsa-miR-3149	1.6884	11.8345	0.01072	0.14175
11014	hsa-miR-214-3p/mmu-miR-214-3p/rno-miR-214-3p	1.7182	8.9916	0.01099	0.14389
169204	hsa-miR-4709-3p	1.6509	12.2926	0.01104	0.14391
169320	hsa-miR-4468	1.7911	11.5738	0.01187	0.14775
11108	hsa-miR-425-3p/mmu-miR-425-3p	0.5879	9.7745	0.01194	0.14775
10306	hsa-miR-146b-5p/mmu-miR-146b-5p/rno-	1.9020	9.3667	0.01196	0.14775

	miR-146b-5p				
148524	hsa-miR-3914	1.5002	8.8473	0.01196	0.14775
147722	hsa-miR-4306	1.5555	12.5408	0.01271	0.15101
27740	hsa-miR-574-5p/mmu-miR-574-5p	1.6121	11.5842	0.01278	0.15122
27545	hsa-miR-373-5p	1.5379	8.5684	0.01295	0.15181
147851	hsa-miR-3201	1.7149	11.2976	0.01430	0.16127
10952	hsa-miR-146a-5p/mmu-miR-146a-5p/rno-miR-146a-5p	2.9263	9.0896	0.01453	0.16127
169137	hsa-miR-4524b-5p	1.8618	9.4566	0.01475	0.16127
10967	hsa-miR-16-5p/mmu-miR-16-5p/rno-miR-16-5p	2.6513	9.7398	0.01481	0.16127
169214	hsa-miR-4638-5p	2.6844	9.2371	0.01488	0.16127
169412	hsa-miR-1260a	0.5010	14.0187	0.01533	0.16272
46732	hsa-miR-1264	0.6418	15.1944	0.01600	0.16393
169203	hsa-miR-5095	0.6622	8.6591	0.01615	0.16416
42542	hsa-miR-589-5p	0.4933	12.6909	0.01628	0.16435
168852	hsa-miR-4764-5p	1.5991	11.3799	0.01644	0.16435
17625	hsa-miR-627-5p	1.5376	8.6427	0.01720	0.16688
168942	hsa-miR-4636	0.5987	11.8286	0.01731	0.16688
148285	hsa-miR-3941	1.5049	11.5263	0.01747	0.16688
148052	hsa-miR-374c-3p/mmu-miR-374c-3p	1.5347	8.9028	0.01777	0.16804
168750	hsa-miR-4645-5p	1.7017	10.2181	0.01787	0.16832
146158	hsa-miR-3202	1.5321	11.3771	0.01942	0.17502
169271	hsa-miR-4784	1.5395	11.2260	0.01942	0.17502
169330	hsa-miR-23b-3p/mmu-miR-23b-3p/rno-miR-23b-3p	2.8403	9.3459	0.01951	0.17515
169228	hsa-miR-4698	1.7014	12.1147	0.02055	0.17877
46788	hsa-miR-1299	1.5393	11.3202	0.02059	0.17877
147845	hsa-miR-3173-3p	1.6119	8.7301	0.02079	0.17895
169409	hsa-miR-4286	0.5329	13.1551	0.02236	0.18777
169260	hsa-miR-4436b-3p	1.5186	10.2751	0.02242	0.18777
148465	hsa-miR-3611	1.6195	12.4081	0.02392	0.19386
169023	hsa-miR-4712-3p	1.5090	12.2238	0.02397	0.19386
42942	hsa-miR-134-5p/mmu-miR-134-5p/rno-miR-134-5p	1.5725	8.2742	0.02637	0.20065
169380	hsa-miR-3124-3p	1.5716	13.5072	0.02685	0.20164
169390	hsa-miR-4800-5p	1.5637	10.1228	0.02689	0.20164
169410	hsa-miR-556-5p	1.6886	8.1373	0.02805	0.20747
10995	hsa-miR-199a-3p/hsa-miR-199b-3p/mmu-miR-199a-3p/mmu-miR-199b-3p/rno-miR-199a-3p	1.8530	8.8890	0.03036	0.21283
145833	hsa-miR-596	1.6949	8.6767	0.03129	0.21541
168926	hsa-miR-4776-5p	1.7153	7.9336	0.03132	0.21541
147616	hsa-miR-4291	1.6127	8.9702	0.03191	0.21541
169226	hsa-miR-4419a	1.5328	8.6299	0.03196	0.21541
42571	hsa-miR-129-1-3p/mmu-miR-129-1-3p	1.6015	9.5756	0.03411	0.22282
42532	hsa-miR-22-5p/mmu-miR-22-5p/rno-miR-22-5p	1.6168	9.7349	0.03594	0.22793
168791	hsa-miR-4785	1.5897	7.6866	0.03644	0.22968

17676	hsa-miR-152-3p/mmu-miR-152-3p/rno-miR-152-3p	1.5294	8.2722	0.03812	0.23620
147755	hsa-miR-378c	1.5557	10.2434	0.03826	0.23620
14280	hsa-miR-367-3p/mmu-miR-367-3p	1.6045	8.1550	0.03996	0.24150
168605	hsa-miR-4653-3p	0.5324	11.2738	0.04082	0.24267
10985	hsa-miR-191-5p/mmu-miR-191-5p/rno-miR-191a-5p	1.6840	9.8605	0.04085	0.24267
169163	hsa-miR-4685-5p	1.5032	8.2110	0.04289	0.24817
169089	hsa-miR-4470	0.4365	11.2768	0.04409	0.25190
145798	hsa-miR-142-5p/mmu-miR-142a-5p/rno-miR-142-5p	2.5513	9.2922	0.04598	0.25719
147376	hsa-miR-3679-5p	0.6507	8.5420	0.04633	0.25755
147828	hsa-miR-3121-3p	1.7425	8.1504	0.04679	0.25904
145678	hsa-miR-150-5p/mmu-miR-150-5p/rno-miR-150-5p	0.5697	11.4748	0.04765	0.26042
168912	hsa-miR-4446-3p	1.5807	8.2624	0.04766	0.26042
17946	hsa-miR-192-3p/mmu-miR-192-3p	1.5010	8.7172	0.04782	0.26043
169183	hsa-miR-4644	1.5991	12.8274	0.04905	0.26285

Supplementary Table S2.
MiRNAs regulated in the <u>heart</u> (left ventricle) of HL hamsters with $p < 0.05$ and fold-change ≥ 1.5, ranked with fold-change.

Gene.ID	Name	Fold-change	Expression level	p-value	FDR
10952	hsa-miR-146a-5p/mmu-miR-146a-5p/rno-miR-146a-5p	1.7947	9.6143	6.84E-05	0.2216
169183	hsa-miR-4644	1.7177	11.3265	1.12E-03	0.6514
11024	hsa-miR-223-3p/mmu-miR-223-3p/rno-miR-223-3p	1.5649	9.8145	1.75E-03	0.6514
42899	hsa-miR-377-5p/mmu-miR-377-5p/rno-miR-377-5p	1.6029	9.3749	1.92E-03	0.6514
10306	hsa-miR-146b-5p/mmu-miR-146b-5p/rno-miR-146b-5p	1.5273	9.7599	2.25E-03	0.6514
42635	hsa-miR-541-3p	1.5682	8.0704	2.66E-03	0.6514
169383	hsa-miR-548ah-5p	1.6620	10.2784	6.53E-03	0.6514
42869	hsa-miR-936	1.7369	9.7293	6.63E-03	0.6514
169061	hsa-miR-548x-3p	1.5276	8.0344	7.74E-03	0.6514
148674	hsa-miR-4321	1.5566	9.2286	0.01058	0.6514
145701	hsa-miR-668-3p/mmu-miR-668-3p/rno-miR-668	1.8989	11.2629	0.01146	0.6514
27544	hsa-miR-363-5p/rno-miR-363-5p	1.5634	10.5207	0.01291	0.6514
42866	hsa-miR-451a/mmu-miR-451a/rno-miR-451-5p	2.0952	12.2352	0.01340	0.6514
42532	hsa-miR-22-5p/mmu-miR-22-5p/rno-miR-22-5p	1.5591	11.1664	0.01532	0.6514
11104	hsa-miR-422a	1.6735	12.5212	0.01648	0.6514
169174	hsa-miR-4709-5p	1.5798	7.9217	0.02171	0.6514
169161	hsa-miR-4489	1.5618	8.7848	0.02223	0.6514
169137	hsa-miR-4524b-5p	1.7029	12.2126	0.02853	0.6514
168948	hsa-miR-4514	1.7659	12.9517	0.03268	0.6514
147545	hsa-miR-3128	1.5303	7.9060	0.03305	0.6514
42865	hsa-miR-181a-5p/mmu-miR-181a-5p/rno-miR-181a-5p	1.5644	11.0346	0.03351	0.6514
168935	hsa-miR-4687-3p	1.5017	9.5456	0.03413	0.6514
169022	hsa-miR-4797-5p	1.5003	12.5195	0.03423	0.6514
169272	hsa-miR-4419b	0.5771	15.4397	0.03523	0.6514
29802	hsa-miR-144-3p/mmu-miR-144-3p/rno-miR-144-3p	2.0341	10.3772	0.04292	0.6514
11149	hsa-miR-515-5p	1.5298	11.0079	0.04313	0.6514

Supplementary Table S3.
MiRNAs regulated in the small intestine (jejunum) of HL hamsters with $p < 0.05$ and fold-change ≥ 1.5, ranked with fold-change.

Gene.ID	Name	Fold-change	Expression level	p-value	FDR
168918	hsa-miR-4491	2.6883	9.6938	8.40E-05	0.1028
169171	hsa-miR-4436b-5p	1.8325	10.7789	1.81E-04	0.1431
42530	hsa-let-7a-2-3p/mmu-let-7a-2-3p/rno-let-7a-2-3p	1.8770	11.7772	2.72E-04	0.1725
168668	hsa-miR-4732-3p	1.7417	12.4472	8.33E-04	0.3303
169387	hsa-miR-5703	1.8483	7.2575	3.75E-03	0.6024
148652	hsa-miR-620	1.7548	9.9678	4.08E-03	0.6053
168579	hsa-miR-5706	1.6763	7.8627	0.01037	0.7623
169096	hsa-miR-4804-3p	1.7583	10.8234	0.01043	0.7623
27533	hsa-miR-320a/mmu-miR-320-3p/rno-miR-320-3p	1.5006	8.9623	0.01241	0.8374
147851	hsa-miR-3201	1.5331	10.7844	0.01277	0.8417
168704	hsa-miR-506-5p	1.7191	9.1296	0.01581	0.8417
42761	hsa-miR-675-5p	0.6560	9.5721	0.01753	0.8417
42926	hsa-miR-92a-1-5p	1.6112	8.1158	0.01875	0.8521
168844	hsa-miR-4532	1.7509	14.0848	0.01984	0.8644
42633	hsa-miR-885-3p	1.8474	8.4214	0.02085	0.8650
14272	hsa-miR-542-3p/mmu-miR-542-3p/rno-miR-542-3p	1.6110	8.7914	0.02448	0.8650
147376	hsa-miR-3679-5p	1.7216	7.2449	0.02516	0.8650
46866	hsa-miR-1321	1.5013	10.7499	0.02865	0.8713
168718	hsa-miR-4684-3p	1.5805	7.7814	0.03309	0.8713
169383	hsa-miR-548ah-5p	1.6413	9.5895	0.03448	0.8713
169170	hsa-miR-4472	0.5576	9.7889	0.03993	0.8713
169228	hsa-miR-4698	2.1992	10.9521	0.04004	0.8713
169393	hsa-miR-4747-5p	0.6573	15.0328	0.04578	0.8742

Supplementary Table S4
Venn diagram data - miRNAs regulated in HL hamsters' tissues; miRNAs were selected by filtered by p value ≤0.05 and FC ≥1.5 or FC≤-1.5.

Names	Total	Elements
**Heart Liver**	**7**	hsa-miR-146a-5p/mmu-miR-146a-5p/rno-miR-146a-5p
		hsa-miR-4644
		hsa-miR-422a
		hsa-miR-22-5p/mmu-miR-22-5p/rno-miR-22-5p
		hsa-miR-146b-5p/mmu-miR-146b-5p/rno-miR-146b-5p
		hsa-miR-377-5p/mmu-miR-377-5p/rno-miR-377-5p
		hsa-miR-4524b-5p
**Intestine Liver**	**8**	hsa-miR-4698
		hsa-miR-4732-3p
		hsa-miR-3201
		hsa-miR-4804-3p
		hsa-let-7a-2-3p/mmu-let-7a-2-3p/rno-let-7a-2-3p
		hsa-miR-4436b-5p
		hsa-miR-620
		hsa-miR-3679-5p
**Heart Intestine**	**1**	hsa-miR-548ah-5p
**Liver**	**118**	hsa-miR-221-5p/mmu-miR-221-5p/rno-miR-221-5p
		hsa-miR-192-3p/mmu-miR-192-3p
		hsa-miR-4305
		hsa-miR-4275
		hsa-miR-665
		hsa-miR-374c-3p/mmu-miR-374c-3p
		hsa-miR-574-5p/mmu-miR-574-5p
		hsa-miR-4306
		hsa-miR-4709-3p
		hsa-miR-4255
		hsa-miR-23c
		hsa-miR-4712-3p
		hsa-miR-3914
		hsa-miR-4279
		hsa-miR-4636
		hsa-miR-374b-3p
		hsa-miR-4455
		hsa-miR-25-5p/mmu-miR-25-5p
		hsa-miR-4764-3p
		hsa-miR-134-5p/mmu-miR-134-5p/rno-miR-134-5p
		hsa-miR-16-5p/mmu-miR-16-5p/rno-miR-16-5p
		hsa-miR-106b-5p/mmu-miR-106b-5p/rno-miR-106b-5p
		hsa-miR-21-5p/mmu-miR-21a-5p/rno-miR-21-5p
		hsa-miR-200b-3p/mmu-miR-200b-3p/rno-miR-200b-3p

		hsa-miR-3940-5p
		hsa-miR-3915
		hsa-miR-1299
		hsa-miR-589-5p
		hsa-miR-32-3p/mmu-miR-32-3p/rno-miR-32-3p
		hsa-miR-1246
		hsa-miR-518a-5p/hsa-miR-527
		hsa-miR-576-5p
		hsa-miR-4470
		hsa-miR-214-3p/mmu-miR-214-3p/rno-miR-214-3p
		hsa-miR-4436b-3p
		hsa-miR-1264
		hsa-miR-4291
		hsa-miR-596
		hsa-miR-4762-5p
		hsa-miR-1184
		hsa-miR-4468
		hsa-miR-199a-3p/hsa-miR-199b-3p/mmu-miR-199a-3p/mmu-miR-199b-3p/rno-miR-199a-3p
		hsa-miR-23a-3p/mmu-miR-23a-3p/rno-miR-23a-3p
		hsa-miR-200a-3p/mmu-miR-200a-3p/rno-miR-200a-3p
		hsa-miR-663a
		hsa-miR-378c
		hsa-miR-4286
		hsa-miR-142-3p/mmu-miR-142a-3p/rno-miR-142-3p
		hsa-miR-4299
		hsa-miR-4421
		hsa-miR-4800-3p
		hsa-miR-3121-3p
		hsa-miR-3941
		hsa-miR-892a
		hsa-miR-423-5p/mmu-miR-423-5p/rno-miR-423-5p
		hsa-miR-367-3p/mmu-miR-367-3p
		hsa-miR-4534
		hsa-miR-4707-5p
		hsa-miR-3611
		hsa-miR-4540
		hsa-miR-4473
		hsa-miR-4456
		hsa-miR-1-5p/mmu-miR-1a-1-5p
		hsa-miR-638
		hsa-miR-183-5p/mmu-miR-183-5p/rno-miR-183-5p
		hsa-miR-1260a
		hsa-miR-4433a-3p
		hsa-miR-3156-3p
		hsa-miR-3591-5p
		hsa-miR-548ar-3p

		hsa-miR-3148
		hsa-miR-373-5p
		hsa-miR-601
		hsa-miR-378d
		hsa-miR-4638-5p
		hsa-miR-4426
		hsa-miR-122-3p/mmu-miR-122-3p
		hsa-miR-4463
		hsa-miR-642b-3p
		hsa-miR-4651
		hsa-miR-2054
		hsa-miR-4419a
		hsa-miR-4784
		hsa-miR-758-5p/mmu-miR-758-5p/rno-miR-758-5p
		hsa-miR-4431
		hsa-miR-191-5p/mmu-miR-191-5p/rno-miR-191a-5p
		hsa-miR-1265
		hsa-miR-3121-5p
		hsa-miR-1255a
		hsa-miR-627-5p
		hsa-miR-4685-5p
		hsa-miR-4657
		hsa-miR-5095
		hsa-miR-23b-3p/mmu-miR-23b-3p/rno-miR-23b-3p
		hsa-miR-4645-5p
		hsa-miR-152-3p/mmu-miR-152-3p/rno-miR-152-3p
		hsa-miR-548an
		hsa-miR-4441
		hsa-miR-129-1-3p/mmu-miR-129-1-3p
		hsa-miR-3173-3p
		hsa-miR-4800-5p
		hsa-miR-3158-5p
		hsa-miR-4446-3p
		hsa-miR-2355-3p
		hsa-miR-3202
		hsa-miR-3149
		hsa-miR-150-5p/mmu-miR-150-5p/rno-miR-150-5p
		hsa-miR-3133
		hsa-miR-378a-3p/mmu-miR-378a-3p/rno-miR-378a-3p
		hsa-miR-4750-5p
		hsa-miR-142-5p/mmu-miR-142a-5p/rno-miR-142-5p
		hsa-miR-4785
		hsa-miR-4653-3p
		hsa-miR-4764-5p
		hsa-miR-4776-5p
		hsa-miR-425-3p/mmu-miR-425-3p

		hsa-miR-3124-3p
		hsa-miR-556-5p
**Heart**	**18**	hsa-miR-363-5p/rno-miR-363-5p
		hsa-miR-668-3p/mmu-miR-668-3p/rno-miR-668
		hsa-miR-144-3p/mmu-miR-144-3p/rno-miR-144-3p
		hsa-miR-4514
		hsa-miR-515-5p
		hsa-miR-4687-3p
		hsa-miR-4321
		hsa-miR-181a-5p/mmu-miR-181a-5p/rno-miR-181a-5p
		hsa-miR-4419b
		hsa-miR-4797-5p
		hsa-miR-223-3p/mmu-miR-223-3p/rno-miR-223-3p
		hsa-miR-541-3p
		hsa-miR-451a/mmu-miR-451a/rno-miR-451-5p
		hsa-miR-4489
		hsa-miR-548x-3p
		hsa-miR-3128
		hsa-miR-4709-5p
		hsa-miR-936
**Intestine**	**14**	hsa-miR-1321
		hsa-miR-4491
		hsa-miR-320a/mmu-miR-320-3p/rno-miR-320-3p
		hsa-miR-675-5p
		hsa-miR-4532
		hsa-miR-506-5p
		hsa-miR-5703
		hsa-miR-4747-5p
		hsa-miR-4684-3p
		hsa-miR-92a-1-5p
		hsa-miR-542-3p/mmu-miR-542-3p/rno-miR-542-3p
		hsa-miR-4472
		hsa-miR-885-3p
		hsa-miR-5706
